# Vertical Ground Reaction Forces in Parkinson’s Disease: A Speed-Matched Comparative Analysis with Healthy Subjects

**DOI:** 10.3390/s24010179

**Published:** 2023-12-28

**Authors:** Marica Giardini, Anna Maria Turcato, Ilaria Arcolin, Stefano Corna, Marco Godi

**Affiliations:** 1 Division of Physical Medicine and Rehabilitation, Istituti Clinici Scientifici Maugeri IRCCS, Institute of Veruno, 28013 Gattico-Veruno, Italy; marica.giardini@icsmaugeri.it (M.G.); stefano.corna@icsmaugeri.it (S.C.); marco.godi@icsmaugeri.it (M.G.); 2 Rehabilitation Department, The Clavadel—The Geoghegan Group, 1 Pit Farm Road, Guildford GU1 2JH, Surrey, UK; anna.t@ggroup.co.uk

**Keywords:** curvilinear walking, gait, gait variability, Parkinson’s disease, plantar pressure, rehabilitation, vertical ground reaction forces

## Abstract

This study aimed to investigate and compare the vertical Ground Reaction Forces (vGRFs) of patients with Parkinson’s Disease (PwPD) and healthy subjects (HS) when the confounding effect of walking speed was absent. Therefore, eighteen PwPD and eighteen age- and linear walking speed-matched HS were recruited. Using plantar pressure insoles, participants walked along linear and curvilinear paths at self-selected speeds. Interestingly, PwPD exhibited similar walking speed to HS during curvilinear trajectories (*p* = 0.48) and similar vGRF during both linear and curvilinear paths. In both groups, vGRF at initial contact and terminal stance was higher during linear walking, while vGRF at mid-stance was higher in curvilinear trajectories. Similarly, the time to peak vGRF at each phase showed no significant group differences. The vGRF timing variability was different between the two groups, particularly at terminal stance (*p* < 0.001). In conclusion, PwPD and HS showed similar modifications in vGRF and a similar reduction in gait speed during curvilinear paths when matched for linear walking speed. This emphasized the importance of considering walking speed when assessing gait dynamics in PwPD. This study also suggests the possibility of the variability of specific temporal measures in differentiating the gait patterns of PwPD versus those of HS, even in the early stages of the disease.

## 1. Introduction

During walking, the human foot generates a ground reaction force (GRF) profile characterized by a distinctive and stereotyped shape. GRFs during gait comprise three components, acting in the antero-posterior direction, in the medio-lateral direction, and in the vertical direction [1]. One of the most distinctive characteristics of human bipedal walking is the two-peaked profile of the vertical component of GRF (vGRF). This profile sets human bipedal walking apart from the gait of any other species of animal [2]. Even monkeys, trained for years to mimic human walking, exhibit structural modifications similar to those of humans but do not display the two force peaks in the vGRF profile during walking [3]. Therefore, this profile represents a unique characteristic of the human species, which is why it has been frequently studied in clinical and rehabilitative contexts. In fact, several authors analyzed the vGRF data to distinguish the gait patterns of healthy subjects (HS) and individuals with neurological diseases [4,5,6,7,8].

The early diagnosis of motor signs is crucial for effective rehabilitative and pharmacological treatment in patients with Parkinson’s Disease (PwPD) [9]. Therefore, the recognition and classification of force patterns are of vital importance in PwPD. However, in studies focusing on vGRF data, PwPD predominantly exhibits a significantly reduced gait speed compared to HS. Such a decrease in speed is associated with a concurrent reduction in the peaks of vGRF [6,7,8,10,11,12].

These findings are in line with the research conducted by Andriacchi et al. [13], who demonstrated a robust association between peaks of vGRFs and gait speed. Furthermore, it is important to highlight that, in many of the aforementioned studies, the measurement of forces typically occurs during linear walking. While straight walking is a common component of daily life, non-straight steps constitute approximately 35–45% of all steps during a typical day [14]. Moreover, the challenges of curvilinear paths are particularly pronounced in PwPD, who commonly face unique gait difficulties due to basal ganglia lesions [15]. During curvilinear paths, differences in forces and muscle activation between the inside and outside legs are influenced by foot placement to counteract centrifugal forces [15]. Analysis in PwPD often shows en-bloc rotations of the body, with affected axial muscle activation and coordination [15].

In light of these observations, the present study aims to analyze and compare the vGRF of PwPD and HS during both linear and curvilinear trajectories, using an insole-based plantar pressure monitoring technique [16]. We can therefore hypothesize that, during straight-line walking, differences in vGRF between PwPD and HS should be minimal when both groups walk at the same speed; conversely, a curvilinear path may highlight some differences in the vGRF within the foot.

## 2. Materials and Methods

### 2.1. Participants

Eighteen patients with mild to moderate PD (mean Hoehn and Yahr (H&Y) 2.1 ± 0.4) [17], aged 71.4 ± 8.0 years, were recruited in the Laboratory of Posture and Movement of the Istituti Clinici Scientifici Maugeri of Veruno (Piedmont, Italy). Eighteen HS were selected and matched for age and for self-selected walking speed along the linear trajectory from a large convenience sample of 329 HS collected among years in the laboratory. For each participant, the medical history was collected, and the same exclusion criteria were screened: postural hypotension or dizziness, orthopedic or arthritic disease known to affect walking, and other neurological disorders. Patients with cognitive impairment (Mini-Mental State Evaluation Test < 24 score [18]) were excluded.

All the included subjects were able to walk without aid in their daily activities, reported no falls in the previous 3 months, and were free from ankle or foot pathologies or other conditions inducing postural instability or movement dysfunction. All PwPD were tested on a stable medication regimen without freezing of gait or deep brain stimulation.

The Institutional Central Ethics Committee (CEC) of the Istituti Clinici Scientifici Maugeri approved this study (approval number 806 CEC), and all participants signed the informed written consent.

### 2.2. Procedures

Both PwPD and HS walked at their self-selected speeds in three different conditions, in random order. These conditions comprised linear and curvilinear paths in both clockwise (CW) and counterclockwise (CCW) directions, at their self-selected speeds. Before data collection, all participants performed a familiarization trial for each condition. Then, during linear walking, participants performed two consecutive 20 m walking trials in the corridor of the rehabilitative ward. During curvilinear trajectories, they completed four trials of 20 m circumference on a radius of 1.2 m, marked with a tape on the floor of a spacious room: two trials were performed in CW and two in CCW directions. Each participant underwent a total of six trials. To ensure data accuracy, the two initial and final gait cycles (corresponding to the first and last four steps) were automatically excluded from data collection by the software. The entire procedure lasted approximately one hour.

### 2.3. Data Collection and Treatment

**Figure 1:**
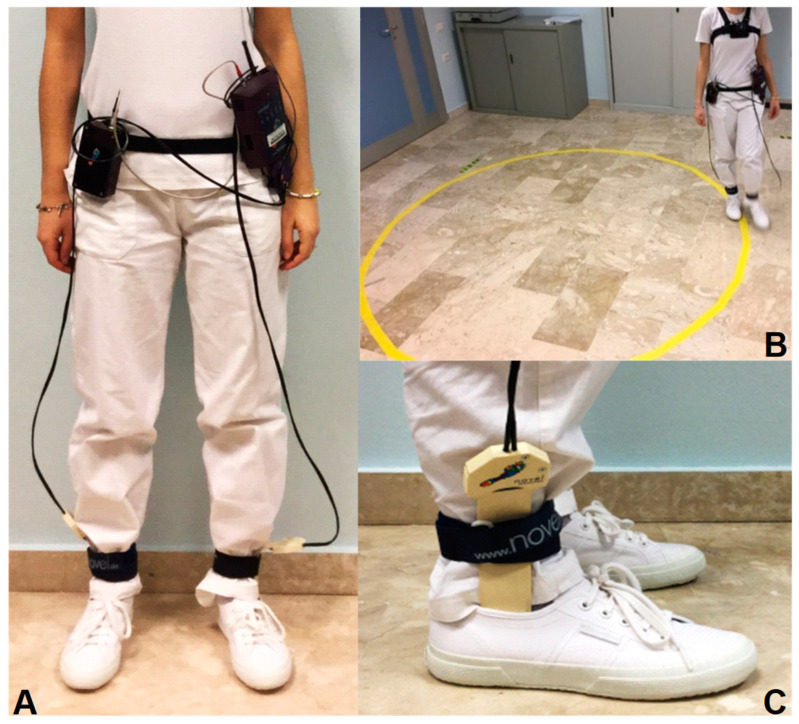
Photographs of the equipment and setup. (**A**) The subject is wearing the belt that connects the Pedar box to the plantar insoles (Pedar-X system, Novel), inserted into Superga shoes. (**B**) The subject completes the curved walking task. (**C**) Close-up of the insole system, showing the strap securing the cable.

For data collection, all participants wore specialized insoles (Pedar-X system, Novel, Germany) paired with shoes (Superga 2750 model, Torino, Italy). The insoles were inserted into both shoes, without socks, and connected to the Pedar box (Figure 1). The high reliability of this Pedar-X system for both linear and curvilinear paths has already been demonstrated [19]. Before starting data collection, the insoles were calibrated using the manufacturer’s proprietary calibration device, Trublu, adhering to the provided manual. Then, a calibration of the insoles was performed for each patient once the insole and the foot were placed in the shoe. This procedure removes artifacts created by the shoes, such as pressure generated by tying the shoe laces. The data were sampled at a frequency of 50 Hz. The variables collected in this study were speed, cadence, stride length, stance duration, vGRFs, time to peak vGRFs, and coefficients of variation (CV) of force and time. The stride length was normalized to height [normalized stride length = (stride length/height)]. The total duration of stance for each subject, defined as the interval time between the heel strike and the toe-off of the same leg, was computed for a more direct comparison between linear and curvilinear paths. Then, these values were used to normalize the times of each peak [Time to peak vGRF (%Stance Time) = (time to peak/stance time) × 100]. The initial peak corresponded broadly to the heel strike and the phase of weight-acceptance (hereafter referred to as initial contact, IC), and the final peak aligned with terminal stance (TS) and toe-off (hereafter referred to as TS). Between the two vGRF peaks, there was a trough; the point at which the vGRF was at its minimum was identified as the foot mid-stance (MS).

For each data sample, the system generated a force value, computed as the sum of the forces registered by the active sensors in each insole. This force value was employed as the vGRF. The vGRF time course was recorded for each trial and subsequently analyzed using the dedicated proprietary software Pedar-X (Expert Version 24). The vGRF was normalized to body weight (BW) converted in Newton [norm %vGRF = (vGRF/BW) × 100] to reduce inter-individual variability [20,21]. Variability of time and force was assessed with the CV, calculated as %CV = (standard deviation (SD)/mean) × 100, which was based on a range of 40–60 steps for each subject and evaluated for each trajectory [22,23].

### 2.4. Statistical Analysis

Results are reported in the table and text as mean ± SD, and in the figures as mean ± standard error (SE).

For continuous and categorical variables, an unpaired Student’s *t*-test or chi-square test were used, respectively, to compare the clinical characteristics of PwPD and HS. When assessing gait variables during a linear trajectory, data obtained from the left and right feet were pooled together. Conversely, for gait variables obtained in curved walking, CW and CCW trials were not analyzed as different conditions since the gait speed was similar in both directions. Feet were defined as “outer” (out) or “inner” (in) depending on their position with respect to the circular trajectory and were considered separately for further analysis.

A test for normality (Shapiro–Wilk) was performed prior to the statistical comparison of the differences in the variables. Gait characteristics (speed, cadence, and normalized stride length), all the vGRFs, and the times to peak vGRF were normally distributed. Thus, for each variable of vGRF and time to peak vGRF, the effects of foot position with respect to the trajectory (linear, out, in) were compared by means of a 2-way repeated-measures analysis of variance (ANOVA) (between groups –PwPD, HS– and within the trajectories –linear, out, in). When ANOVA gave a significant result, the Tukey test was used for post-hoc comparisons. The Bonferroni correction for multiple comparisons was applied, establishing a statistical significance level of *p* < 0.004. On the contrary, the distribution of variability variables for both the vGRF and the times to peak vGRF proved to be non-normal. Therefore, the Mann–Whitney U test was applied for comparison between groups. The Friedman test was then used to assess differences in variables among the three gait trajectories. When the Friedman test result was significant, a post-hoc Wilcoxon test was performed after Bonferroni correction.

The software program STATISTICA (StatSoft, version 12.0) was used for the analyses.

## 3. Results

Table 1 shows the clinical characteristics of PwPD and HS. Each group was balanced for sex (*p* > 0.50 within each group). Groups were similar for age, weight, height (*p* > 0.05), and sex proportion (*p* = 0.50). The mean disease duration was about 9 years, the mean H&Y stage was 2.1, and the mean Unified Parkinson’s Disease Rating Scale (UPDRS) motor section score was about 18. The mean asymmetry score, defined as the difference between the UPDRS scores of the two sides (higher minus lower score), was calculated but not reported, since all patients except one had asymmetry scores < 5, i.e., not asymmetric according to Uitti et al. [24].

### 3.1. Gait Variables

Although participants were matched for linear walking speed, both walking speed (PwPD: 0.86 ± 0.17 m/s; HS: 0.90 ± 0.19 m/s) and cadence (PwPD: 107 ± 12 steps/min; HS: 104 ± 11 steps/min), assessed during curvilinear trajectories, were similar between groups (*p* = 0.48 and *p* = 0.49, respectively). The only detectable difference between groups was in normalized stride length (PwPD: 0.58 ± 0.08 m; HS: 0.62 ± 0.09 m; *p* < 0.05).

### 3.2. Ground Reaction Force in the Stance Phase

**Figure 2:**
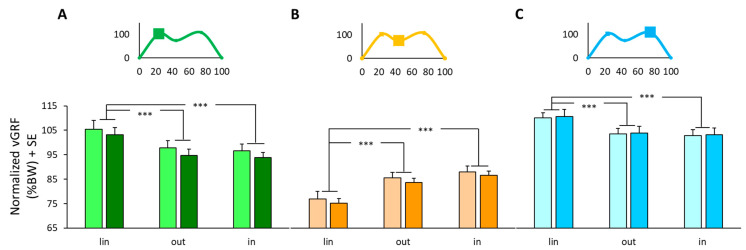
The average of normalized vGRF measured during a linear (lin) trajectory is the average of right and left foot values and curvilinear trajectories in the outer (out) and inner (in) feet. Lighter colors identified PwPD, while darker colors represented HS. Values are obtained from the peak of vGRF at initial contact (**A**, green colors), at mid-stance (**B**, orange colors), and at terminal stance (**C**, light-blue colors). Insets at the top show an example of a vGRF profile with representative peaks and a low phase. BW, body weight; SE, standard error; vGRF, vertical ground reaction force. ***, *p* < 0.001.

At IC (Figure 2A), a two-way ANOVA showed no difference in vGRF between groups (ANOVA, *F*(1,34) = 0.48, *p* = 0.50). On the contrary, a significant effect was found for trajectories (ANOVA, *F*(2,68) = 45.59, *p* < 0.001); the vGRF was larger in linear walking compared to both curvilinear trajectories (Tukey post-hoc, *p* < 0.001). No interaction was found between groups and trajectories (ANOVA, *F*(2,68) = 0.07, *p* = 0.93).

At MS (Figure 2B), a two-way ANOVA showed that there was no difference in vGRF between groups (ANOVA, *F*(1,34) = 0.32, *p* = 0.58). On the contrary, a significant effect was found for trajectories (ANOVA, *F*(2,68) = 111.54, *p* < 0.001); the vGRF was larger during curvilinear paths compared to linear paths (Tukey post-hoc, *p* < 0.001). No interaction was found between groups and trajectories (ANOVA, *F*(2,68) = 0.07, *p* = 0.93).

At TS (Figure 2C), a two-way ANOVA showed that there was no difference in vGRF between groups (ANOVA, *F*(1,34) = 0.02, *p* = 0.90). On the contrary, a significant effect was found for trajectories (ANOVA, *F*(2,68) = 52.59, *p* < 0.001); the vGRF was larger in linear walking compared to both curvilinear trajectories (Tukey post-hoc, *p* < 0.001). No interaction was found between groups and trajectories (ANOVA, *F*(2,68) = 0.01, *p* = 0.99).

### 3.3. Time to Peak Ground Reaction Force

**Figure 3:**
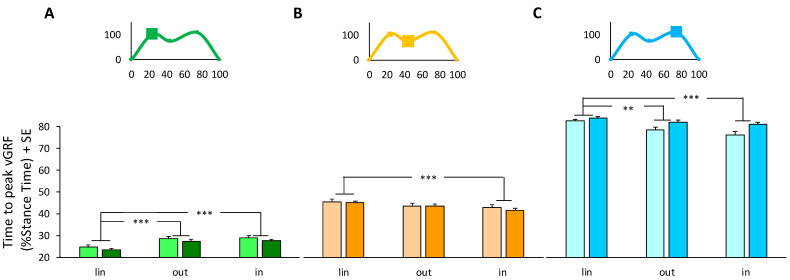
Average of the time to peak vGRF measured during the linear (lin) trajectory, as the average of right and left foot values, and curvilinear trajectories in the outer (out) and inner (in) foot. Lighter colors identified PwPD, while darker colors represented HS. Values are obtained from the time to peak of vGRF at initial contact (**A**, green colors), at mid-stance (**B**, orange colors), and at terminal stance (**C**, light-blue colors). Insets at the top show an example of a vGRF profile with representative peaks and a low phase. SE, standard error; vGRF, vertical ground reaction force. **, *p* < 0.004; ***, *p* < 0.001.

At IC (Figure 3A), a two-way ANOVA showed that there was no difference in the time to peak vGRF between groups (ANOVA, *F*(1,34) = 1.24, *p* = 0.27). On the contrary, a significant effect was found for trajectories (ANOVA, *F*(2,68) = 48.06, *p* < 0.001); the time to peak was earlier in linear walking compared to both curvilinear trajectories (Tukey post-hoc, *p* < 0.0005). No interaction was found between groups and trajectories (ANOVA, *F*(2,68) = 0.02, *p* = 0.98).

At MS (Figure 3B), a two-way ANOVA showed that there was no difference in the time to peak vGRF between groups (ANOVA, *F*(1,34) = 0.20, *p* = 0.66). On the contrary, a significant effect was found for trajectories (ANOVA, *F*(2,68) = 17.55, *p* < 0.001); the time to peak was delayed in linear walking compared to the inner foot in a curvilinear trajectory (Tukey post-hoc, *p* < 0.001). No interaction was found between groups and trajectories (ANOVA, *F*(2,68) = 0.43, *p* = 0.65).

At TS (Figure 3C), a two-way ANOVA showed that there was no difference in time to peak vGRF between groups (ANOVA, *F*(1,34) = 8.22, *p* = 0.007). On the contrary, a significant effect was found for trajectories (ANOVA, *F*(2,68) = 14.28, *p* < 0.001); the time to peak was delayed in linear walking compared to the inner foot (Tukey post-hoc, *p* < 0.001) and outer foot (Tukey post-hoc, *p* < 0.004). No interaction was found between groups and trajectories (ANOVA, *F*(2,68) = 1.93, *p* = 0.15).

### 3.4. Coefficient of Variability

#### 3.4.1. Variability of vGRF

At the IC and MS, the variability of vGRF showed the same behavior. The mean %CV of the vGRF was different in the trajectories (at IC, Friedman’s ANOVA, ꭓ^2^(2,36) = 16.72, *p* < 0.001; at MS, Friedman’s ANOVA, ꭓ^2^(2,36) = 15.72, *p* < 0.001). Post-hoc analysis showed that variability was increased in the outer foot compared to the inner foot (both for IC and MS, Wilcoxon matched-pairs test, *p* < 0.001). The %CV between groups in different trajectories was similar (at IC, Mann–Whitney U test, at least *p* = 0.53; at MS, Mann–Whitney U test, at least *p* = 0.70). At TS, the mean %CV of the vGRF was different in the trajectories (Friedman’s ANOVA, ꭓ^2^(2,36) = 31.72, *p* < 0.001). Post-hoc analysis showed that variability was higher in curved walking compared to the linear one, both in the inner (Wilcoxon matched-pairs test, *p* < 0.001) and outer trajectories (Wilcoxon matched-pairs test, *p* < 0.001). The %CV between groups was similar (Mann–Whitney U test, at least *p* = 0.34).

#### 3.4.2. Variability of Time to Peak vGRF

At IC, the mean %CV of the time to peak vGRF was different in the trajectories (Friedman’s ANOVA, ꭓ^2^(2,36) = 29.56, *p* < 0.001). Post-hoc analysis showed that variability was increased in curved trajectories, both for the inner (Wilcoxon matched-pairs test, *p* < 0.001) and outer foot (Wilcoxon matched-pairs test, *p* < 0.001) compared to linear walking. The %CV between groups was similar (Mann–Whitney U test, at least *p* = 0.32). Similarly, at TS, the mean %CV of the time to peak vGRF was different in the trajectories (Friedman’s ANOVA, ꭓ^2^(2,36) = 45.39, *p* < 0.001). Post-hoc analysis showed that variability was increased in curved trajectories, both for the inner (Wilcoxon matched-pairs test, *p* < 0.001) and outer foot (Wilcoxon matched-pairs test, *p* < 0.001) compared to linear walking. In the inner foot trajectory, PwPD showed a higher %CV of the time to peak vGRF compared to HS (Mann–Whitney U test, *p* < 0.001). At MS, the mean %CV of the time to peak vGRF was similar in the trajectories (Friedman’s ANOVA, ꭓ^2^(2,36) = 2.72, *p* = 0.26), without difference in %CV between groups (Mann–Whitney U test, at least *p* = 0.09).

## 4. Discussion

The current literature utilizes vGRF to differentiate PwPD in the early stages of disease from HS [6,8,25,26,27,28]. Although the time-domain pattern of vGRF during the stance was found to be similar in PwPD and HS [26,29], specific characteristics, such as the peak value, differed significantly [6,7,10]. Consequently, recent studies have increasingly employed machine learning techniques for the diagnosis of PD and for monitoring the effectiveness of pharmacological and rehabilitative treatment [6,28,30].

The present study, however, challenges these findings by revealing the absence of significant differences in the vGRF between PwPD and HS. Notably, the differences in vGRF previously observed were studied in groups with incomparable walking speeds. Some authors found a significantly reduced peak vGRF in PwPD compared to HS when the gait speed was approximately 20% higher in the control group [31,32,33]. Conversely, other studies indicated that PwPD walking at their faster pace had a higher contact force of about 5% [26].

These contrasting examples highlight how the vGRF modulates according to walking speed. While this notion has been acknowledged for years [34], it is often overlooked in studies comparing PwPD and HS, where walking speed differs. Authors tend to highlight differences in vGRF without considering that these distinctions may arise from altered walking speed in patients compared to control subjects and not from the pathology itself. This led to our current study, aiming to eliminate the confounding effect of speed by matching subjects from both groups based on their spontaneous walking speed. Our work emphasizes the importance of considering speed in assessing gait dynamics in PD, aligning with the proposal by Patoz et al. [29].

Our results find support in several studies demonstrating that, at speed-matched, the first peak of the stance phase does not vary with age or gait condition. For instance, Nilsson and Thorstensson [32] showed that the vGRF peak of initial contact remains consistent between walking and running at the same speed, up to a maximum of 9 km per hour. Even minor variations in hip or knee kinematics, common in healthy older adults, are insufficient to induce significant changes in vGRF [35]. Only substantial variations in lower limb kinematics, such as when HS are asked to walk stooped forward [36] or in chimpanzee-like bipedal walking made by humans [37], could effectively influence GRF.

In our study, vGRF remained comparable between PwPD and HS even during curvilinear paths, where both groups experienced a similar reduction in speed. This aligns with previous research indicating a moderate decrease in walking speed on curved paths, influenced by turn angles [15]. Our findings affirm a subtle reduction in walking speed during curvilinear paths, consistent with observations in various populations, including young subjects, the elderly, and patients with different diseases [38,39]. This reduction in speed corresponds to a decrease in peak vGRF, resembling the vGRF reduction observed in young subjects following similar trajectories [40].

It is well established that the curvilinear path engages unique neural processes associated with cognitive flexibility and set-shifting mechanisms. This is manifested by an increased duration of double support and a decreased duration of single support [41], an adaptive strategy commonly used by individuals to enhance postural stability during turning [39,42,43]. These temporal adjustments are more pronounced in PwPD compared to HS [29,44]. However, our study revealed no significant difference in the time to peaks between PwPD and HS in both linear and curvilinear paths. These findings confirmed the results of Patoz et al. [29], where PwPD and HS exhibited similar self-selected walking speeds. In contrast, our results seemingly contradict prior research indicating that the peaks of the vGRF in PwPD generally occur later compared to HS [26,45], suggesting that these differences are likely influenced by disparities in self-selected walking speed.

Variability in stance or swing duration has consistently been associated with the risk of falling in various populations [44,46]. Notably, when compared with age-matched controls, PwPD increased timing variability in temporal measures [26,29,31]. Also in our results, although the confounding effect of walking speed was absent, a higher variability in the time of the second peak of vGRF was found in curved paths. This aspect, unlike other variables, suggests the possible ability of variability measures to distinguish between PwPD and HS, even in the early stages of PD. Therefore, the timing variability seems to be an important aspect to consider when classifying PwPD [7].

### Limitations and Future Directions

Although some authors suggest evaluating PwPD in the off-phase [47], when the characteristics of the disease that are not compensated by pharmacological therapy may emerge [48], we deliberately chose to assess the gait of well-compensated and highly performing patients. This decision allowed a more direct comparison of their gait performance with that of HS; on the contrary, due to the reduction in gait speed of PwPD in the off-phase, it would have been hard to match PwPD in the off-phase with HS of the same age.

Some researchers have raised concerns about the use of insole systems to differentiate gait characteristics between PwPD and HS based on vGRF magnitude. For instance, Barnett et al. [49] showed that vGRF measurements obtained with insole systems were less valid than those obtained with a force platform due to noise reduction thresholds. Other studies noted a delay in data acquisition during specific phases of the gait cycle when using insoles [50]. Therefore, it is advisable to compare the vGRFs collected with the same instruments. Since our study used the same insole system for both PwPD and HS, we feel confident about the validity of our results.

Future studies should assess the application of this insole-based plantar pressure monitoring technique to other diseases, comparing their results to a control group matched for gait speed. This would allow determining whether the characteristics of human vGRF depend solely on gait speed and whether there are underlying diseases that can modify them. Finally, specifically for PwPD, it would be useful to explore other assessment tools that may highlight differences in their gait patterns compared to HS.

## Figures and Tables

**Table 1:** The clinical characteristics and linear speed of PwPD matched those of HS.

	PwPD									HS				
	Sex	Age (years)	Weight (kg)	Height (cm)	H&Y (stage)	Duration (years)	UPDRS III (score)	Side of Onset	Gait Speed (m/s)	Sex	Age (years)	Weight (kg)	Height (cm)	Gait Speed (m/s)
1	F	80	69	160	2.5	13	24	R	0.72	F	69	70	159	0.69
2	M	76	85	175	2	6	21	L	0.91	M	80	74	161	0.87
3	F	71	65	165	2	11	22	L	0.98	F	74	65	165	1.02
4	M	73	100	174	2	7	17	R	1.05	M	67	69	170	1.07
5	F	79	61	163	2.5	11	15	L	1.06	F	72	67	160	1.14
6	F	70	60	160	2.5	9	18	R	1.12	M	84	74	167	1.15
7	M	59	74	170	2	11	19	L	1.18	M	69	60	160	1.16
8	F	69	62	168	2.5	11	20	R	1.22	F	65	60	167	1.18
9	M	76	92	165	2.5	14	26	L	1.22	F	83	84	170	1.18
10	F	62	60	157	1.5	5	9	L	1.23	M	89	70	162	1.20
11	M	79	53	170	2.5	6	22	L	1.25	M	74	75	167	1.24
12	M	75	83	178	2	9	15	R	1.30	F	75	60	153	1.31
13	M	73	92	172	2.5	12	27	L	1.33	F	67	70	165	1.33
14	F	51	90	173	2	10	17	L	1.33	F	70	59	150	1.34
15	M	81	75	167	2.5	8	20	R	1.45	F	63	60	168	1.50
16	M	72	63	170	1.5	5	11	L	1.52	F	60	62	160	1.52
17	M	64	76	162	2.5	4	14	R	1.53	M	75	81	177	1.60
18	F	75	47	163	1	3	9	L	1.58	M	73	65	164	1.63
**MEAN**	**10M; 8F**	**71.4**	**72.6**	**167.3**	**2.1**	**8.6**	**18.1**	**7R;** **11L**	**1.22**	**8M; 10F**	**72.7**	**68.1**	**163.6**	**1.23**
*SD*		*8.0*	*15.1*	*5.9*	*0.4*	*3.2*	*5.3*		*0.23*		*7.6*	*7.5*	*6.4*	*0.24*

Abbreviations: F, female; HS, healthy subjects; H&Y, Hoehn and Yahr; L, left; M, male; PwPD, patients with Parkinson’s Disease; R, right; SD, Standard Deviation; UPDRS III, Unified Parkinson’s Disease Rating Scale, motor section III. Bold: highlight the mean data with respect to the data of each single subject. Italics represent the standard deviation of the mean.

## Data Availability

The data presented in this study are available on request from the corresponding author. The data are not publicly available due to privacy restrictions.
